# Orthodontic Treatment of a Case With Palatally Impacted Canine and Missing Molars: A Case Report

**DOI:** 10.7759/cureus.24741

**Published:** 2022-05-04

**Authors:** Kinjal J Mavani, Mohit J Jain, Vikram Pai, Vijay Naik

**Affiliations:** 1 Department of Orthodontics and Dentofacial Orthopaedics, KM Shah Dental College and Hospital, Sumandeep Vidyapeeth, Waghodia, IND; 2 Department of Orthopaedics, Smt. BK Shah Medical Institute and Dhiraj Hospital, Sumandeep Vidyapeeth, Waghodia, IND; 3 Department of Orthodontics and Dentofacial Orthopaedics, Maratha Mandal Dental College, Belgavi, IND

**Keywords:** balanced occlusion, interdisciplinary care, orthodontic treatment, missing molars, palatally impacted canine

## Abstract

Interdisciplinary care provides a comprehensive method for the management of impacted teeth. Careful planning is necessary to achieve the desired treatment goals. This article highlights the importance of diagnosis as well as adequate treatment planning for the eruption of impacted teeth and the management of missing molars to achieve a balanced occlusion in circumstances when an ideal occlusion cannot be achieved. Here, we have presented a case report of an impacted maxillary canine with lost molars of a 15-year-old female patient.

## Introduction

Palatally impacted canines are considered one of the most difficult cases to be handled by an orthodontist. It requires a multidisciplinary approach involving the orthodontist and an oral surgeon [1]. Primary reasons for impacted teeth are genetics endocrine deficiency, irradiation, palatal clefts, developmental abnormalities, dento-maxillary disharmony, late or missing root development, growth disharmony between pre‑maxilla and maxilla, and transverse growth deficiency of the anterior maxilla [2]. This condition requires the close teamwork of the oral surgeon and the orthodontist-initially to achieve access to the impacted tooth and then to use precise biomechanics to place the tooth in its proper place. Also, early loss of molars in an adolescent patient requires restoration of function and esthetics. Consequently, treatment duration can be extended when malocclusion is complex due to an impacted canine, missing molars, anterior and posterior crossbite, and crowding. This case report describes the interdisciplinary management of one such case.

## Case presentation

Case history

**Figure 1:**
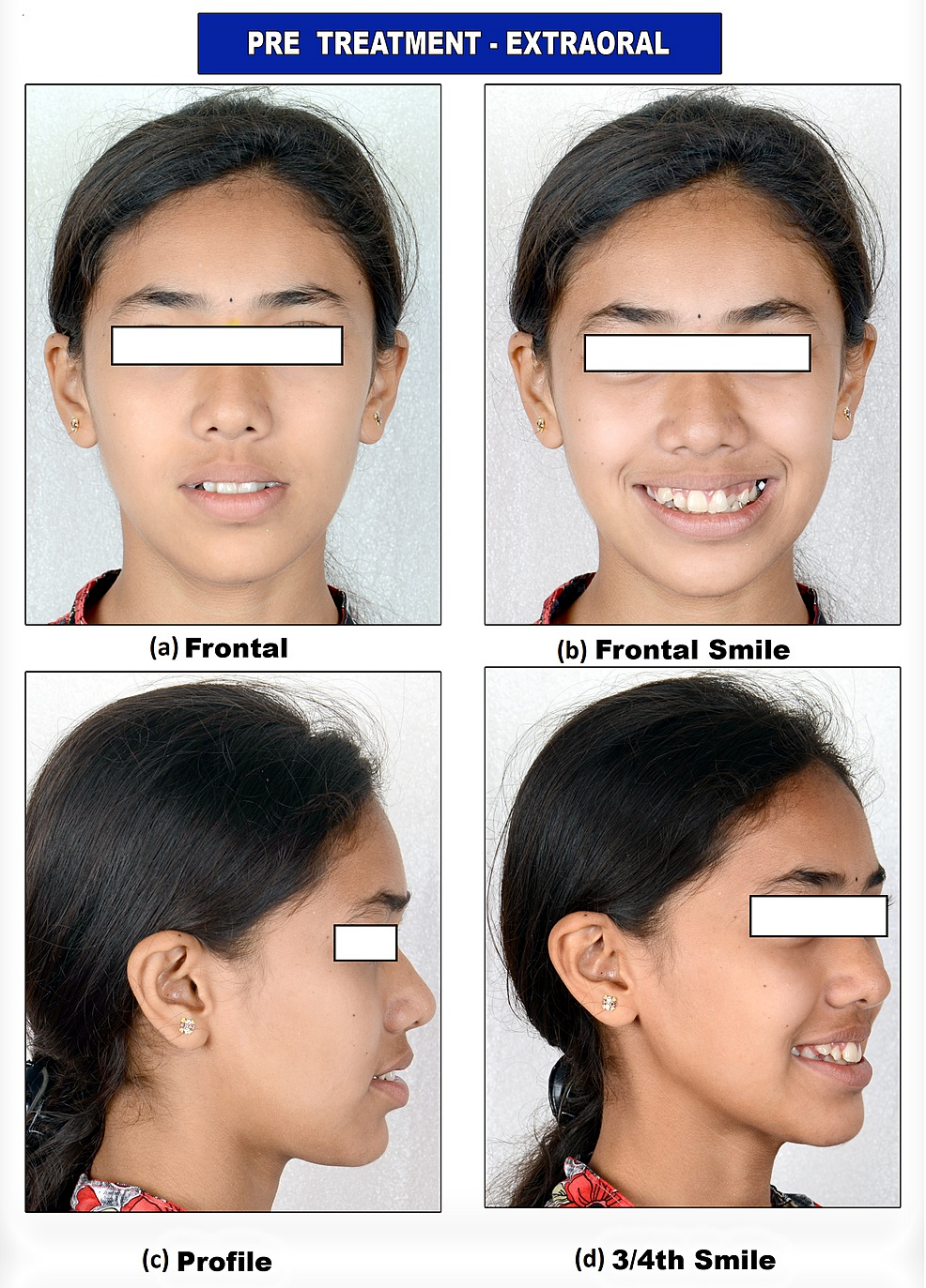
Pre-treatment extraoral photograph views

A 15-year-old female presented to an academic dental institute with the chief complaint of irregularly and forwardly placed teeth in the upper and lower anterior regions. She had a mesoprosopic facial form with a mildly straight profile and a non-consonant smile (Figure 1).

**Figure 2:**
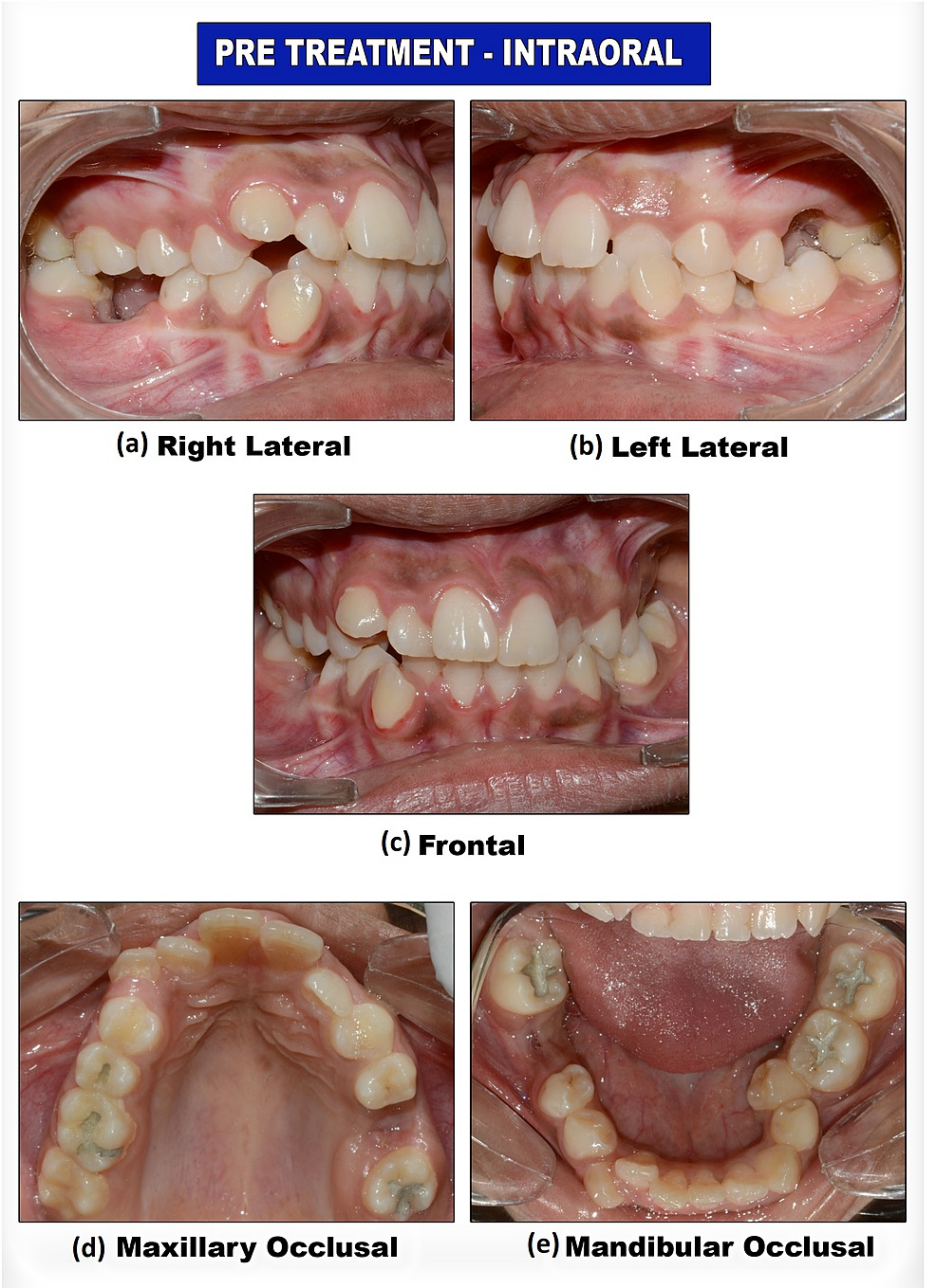
Pre-treatment intraoral photograph views

An intraoral examination (Figure 2) revealed an absent 26 and 46.

They were extracted as they were unrestorable due to carious destruction. A missing 23 with a palatal bulge was seen. An anterior crossbite was present with 22 and a posterior crossbite with 36 and 37. Labially placed 13, 43, and lingually placed 35, 42. A midline shift to the left by 3 mm was seen between the upper dental midline and the facial midline. A mild prominence on the lower lip was present. Radiographic examination revealed a palatally impacted 23 with a mesial inclination of 30 degrees. Oral hygiene was poor. Model analysis revealed a space deficiency of 8 mm in the maxilla and 10 mm in the mandible.

Radiographic examination

**Figure 3:**
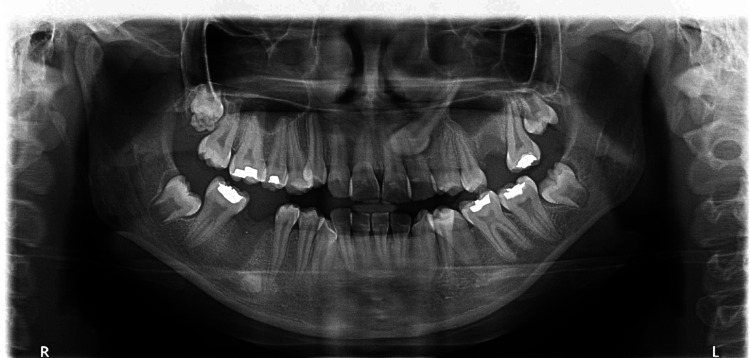
Pre-treatment orthopantomagram radiograph

A panoramic examination revealed a palatally impacted maxillary canine in the left quadrant (Figure 3).

**Figure 4:**
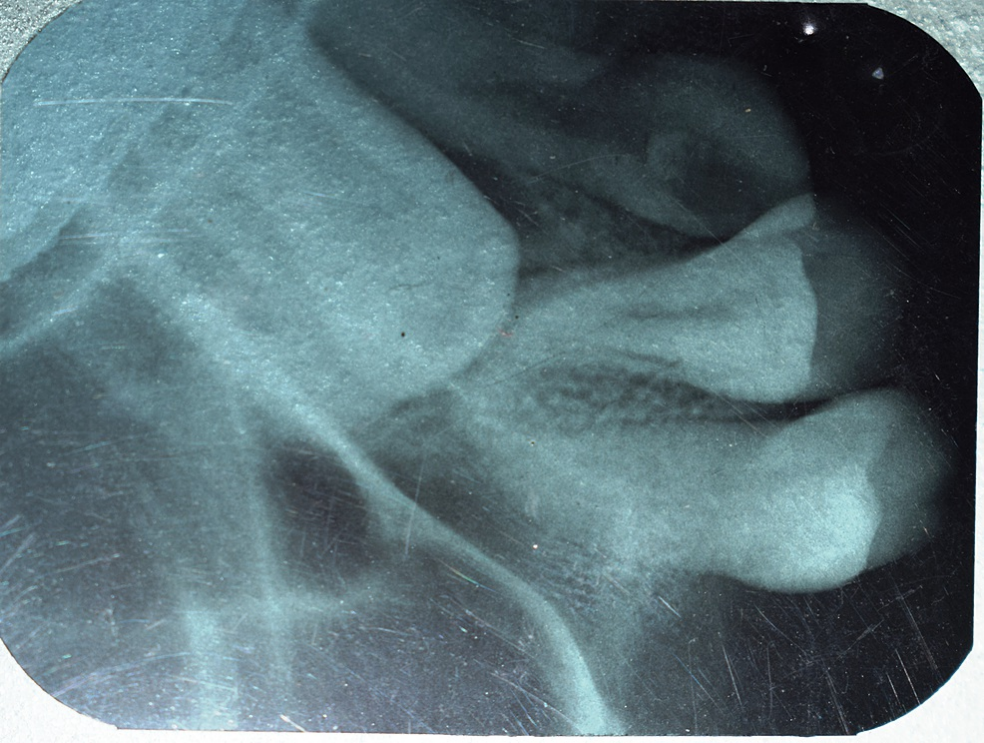
IOPA radiograph with palatally impacted 23 IOPA: intra-oral peri-apical

**Figure 5:**
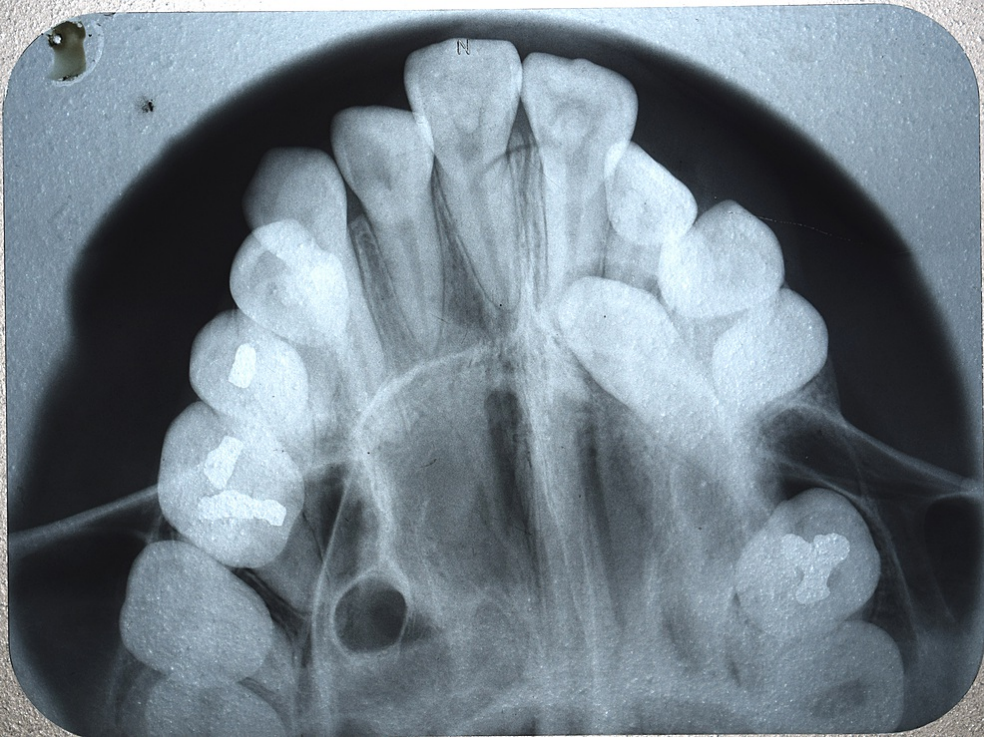
Occlusal radiograph with palatally impacted 23

Erupting third molars were seen in all four quadrants. An intra-oral peri-apical (IOPA) radiograph (Figure 4) and an occlusal radiograph (Figure 5) revealed a palatally impacted 23.

**Figure 6:**
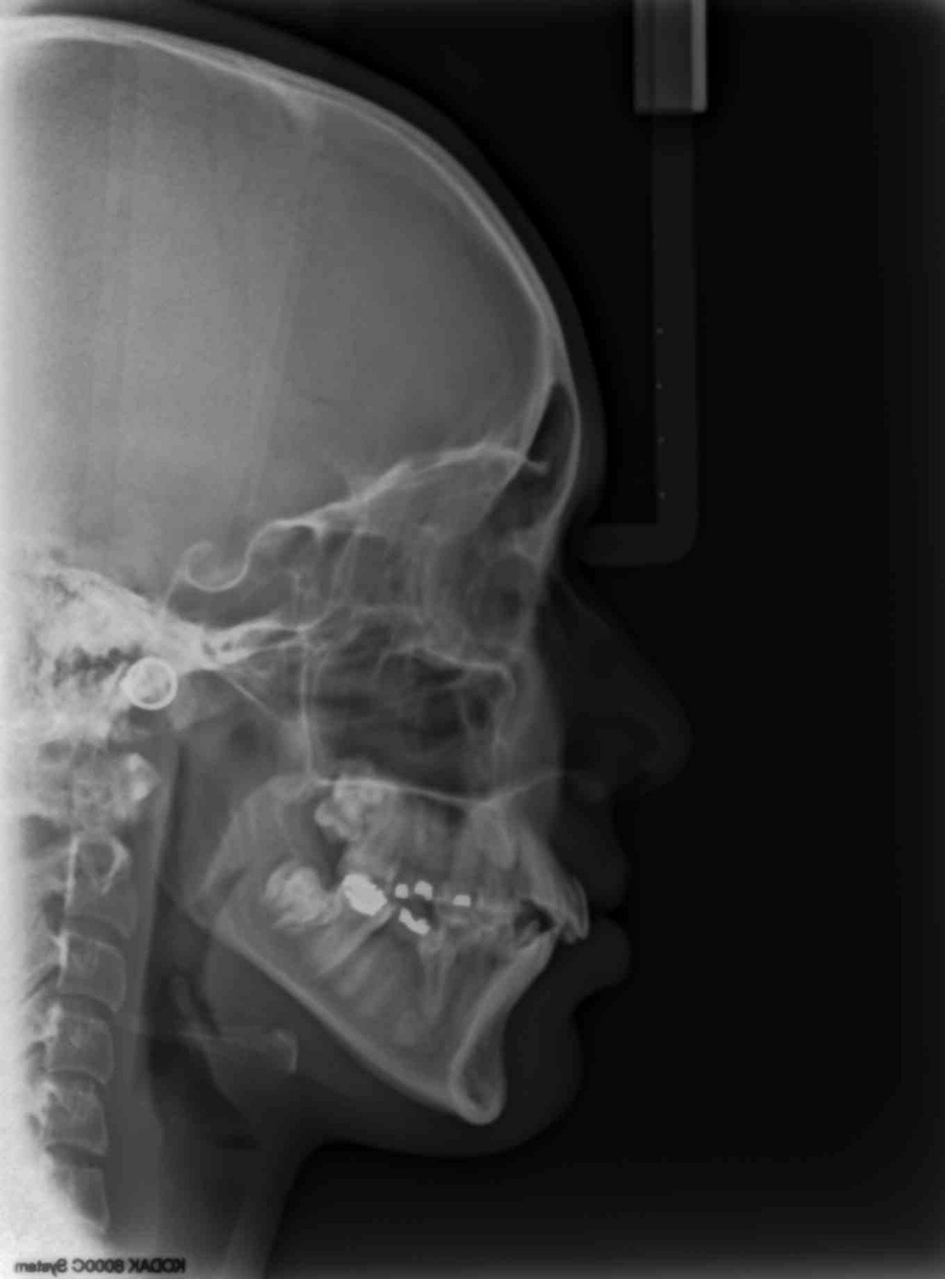
Pre-treatment cephalogram

Cephalometric analysis revealed a class III skeletal base with a vertical growth pattern (Figure 6).

Pre-treatment cephalometric analysis by Steiner's skeletal and dental methods gave the values as listed in Table 1.

**Table 1:** Pre and post-treatment cephalometric values SNA: Sella-Nasion to B point angle, SNB: Sella-Nasion to B point angle, ANB: A to B point angle, FMA: Frankfort-Mandibular angle, IMPA: incisor mandibular plane angle

Variables	Mean	Pre-treatment	Post-treatment	Difference
Maxilla to cranium				
SNA angle	82±2°	73°	75°	2°
Mandible to cranium				
SNB angle	80±2°	75°	76°	1°
Maxilla to mandible				
ANB angle	2±2°	−2°	−2°	0
Wits (mm)	0	−11 mm	−10 mm	−1 mm
Vertical relationship				
Y-axis angle	53–66°	63°	63°	0°
Facial axis angle	90°	93°	94°	1°
FMA angle	25°	42°	38°	4°
Occlusal to SN	23°	33°	25°	8°
Maxillary dental				
UI to NA (angle)	22°	38°	30°	8°
UI to NA (mm)	4 mm	17 mm	13 mm	4mm
UI to SN (angle)	102±2°	110°	102°	6°
Mandibular dental				
LI to NB (angle)	25°	31°	28°	3°
LI to NB (mm)	4 mm	10 mm	9 mm	1 mm
IMPA (angle)	90±5°	91°	86°	5°
Maxilla to mandible (dental)				
UI to LI (angle)	130°	114°	123°	9°

Treatment objectives

It was aimed to achieve proper alignment of the teeth, bringing the impacted canine into the arch, along with achieving class I canine relations bilaterally, satisfactory occlusion, closure of spaces present, ideal overjet, overbite, and coincident midlines. A concordant smile, as well as optimal facial esthetics and balance were desired.

Treatment alternatives

One of the treatment options would have been non-extraction with proximal stripping to gain a minor amount of space while utilizing all of the existing spaces available. This would have had the advantage of avoiding extractions in an already compromised dentition. However, the midline, which is shifted to the left, and the protrusion of the upper anterior would not have been corrected. Thus, the treatment plan selected included extraction of 14 and 35 followed by fixed mechanotherapy with a 0.022-slot MBT. In the upper arch, alignment was planned, followed by space creation for the impacted 23. To create space, individual retraction with 34 and 35, using TADS traction. Following space creation, surgical exposure of 23, and later bringing it into the arch using traction mechanics in the lower arch, the goal was to achieve alignment of the anteriors with decrowding. TADS was used to close the remaining spaces with 47 molar protraction. The retention planned was a lingually bonded retainer for both arches. In this case, considering the treatment objectives, 14 was extracted as the maxillary dental midline was shifted to the left, and also space of almost 8 mm was required for the impacted canine and also for retraction of the anteriors. In the mandibular arch, 45 was selected to be extracted as space was required to relieve crowding and also due to its position in the arch. Molar protraction was planned with 47 for the space left after decrowding of anteriors, as the space was too minimal for any prosthesis. Also, molar protraction would have created space for erupting the third molar, and a natural tooth could be the best replacement for any missing tooth. This would reduce the burden on the patient in terms of the cost of an additional prosthesis.

Treatment progress

**Figure 7:**
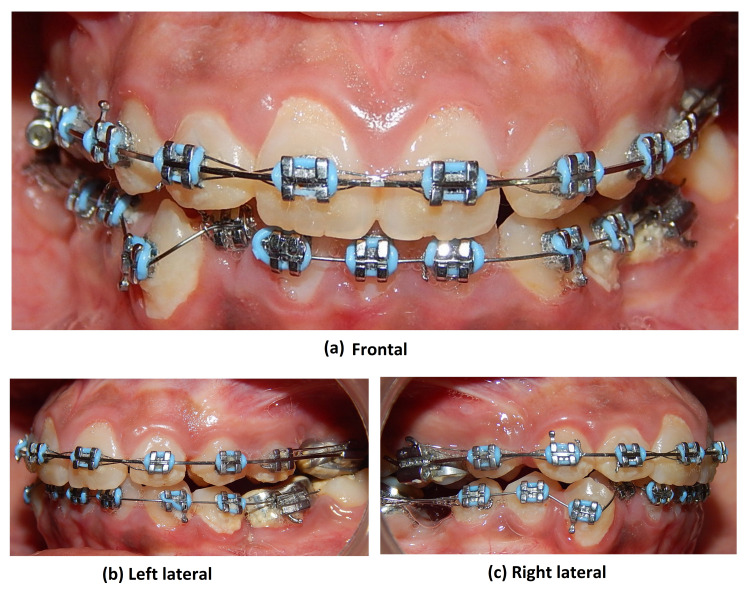
Intraoral photograph views showing alignment with upper arch after TAD supported molar protraction (27) to close the existing space of missing 26

Therapeutic extractions of 14 and 35 as a part of the treatment protocol were done by an oral surgeon. Intraoral strap up was done in both arches. Alignment was started with 0.012 Nickel-titanium wire (Figure 7).

**Figure 8:**
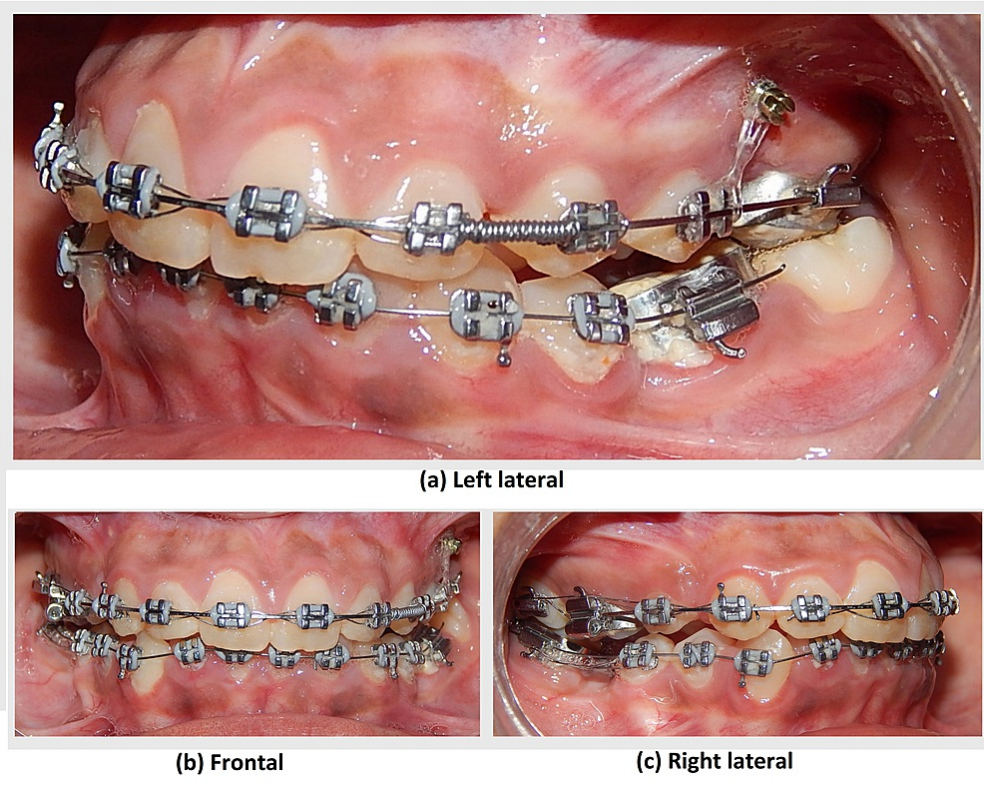
Intraoral photograph views showing canine emergence in the oral cavity and couple force applied for derotation

After four months of aligning at 0.019 × 0.025”, stainless stage temporary anchorage devices (TAD) were placed distal to 25 (Figure 8).

**Figure 9:**
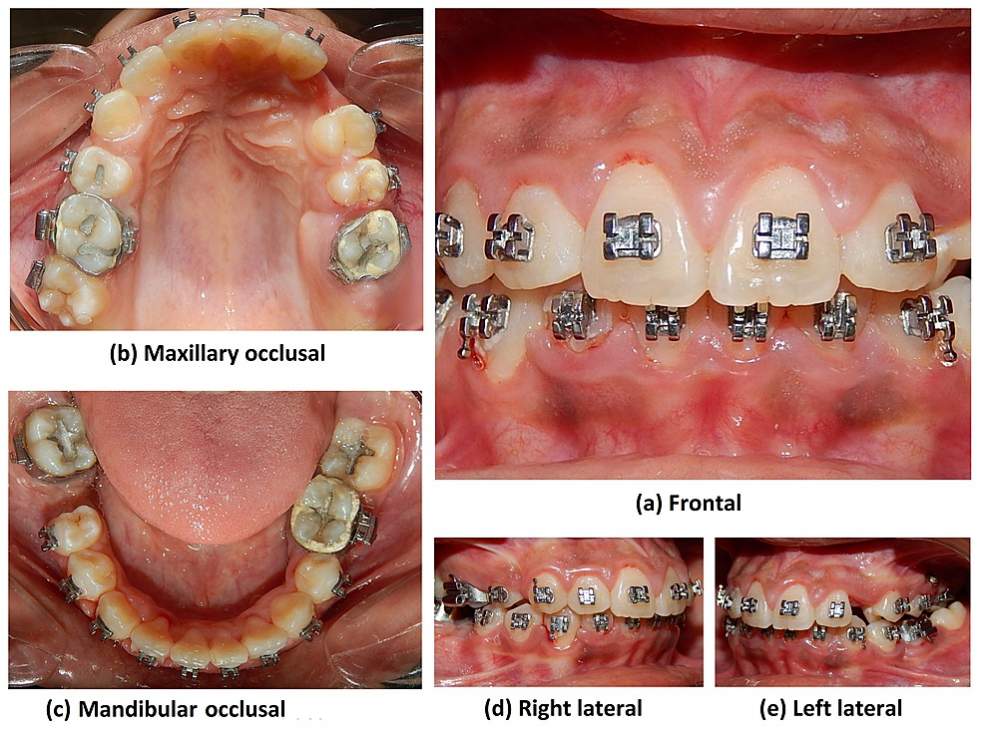
Intraoral photograph views post-alignment and space creation by open coil spring between 22 and 24

The individual retraction was started with 25 and 24 using TADS. Also, an open coil spring was placed between 22 and 24 (Figure 9).

**Figure 10:**
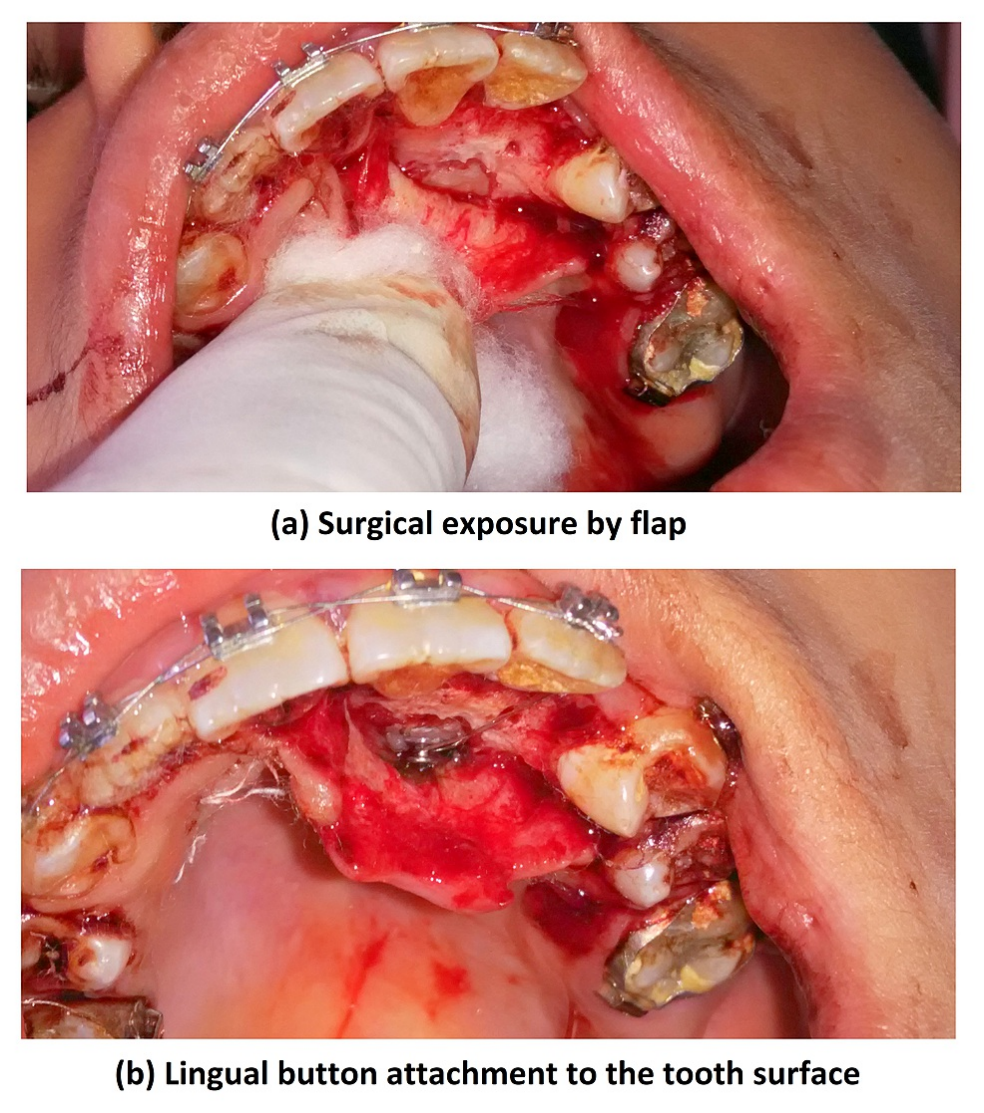
Intraoral photograph views showing surgical exposure with 23

Both of these methods were done with the intention of creating space in-between for the impacted canine. Once space was created between 22 and 24, surgical exposure of the impacted 23 was done in the Department of Oral and Maxillofacial Surgery. A closed eruption technique was selected, a lingual button was bonded to the tooth as an attachment on the tooth surface, and the flap was sutured back (Figure 10).

**Figure 11:**
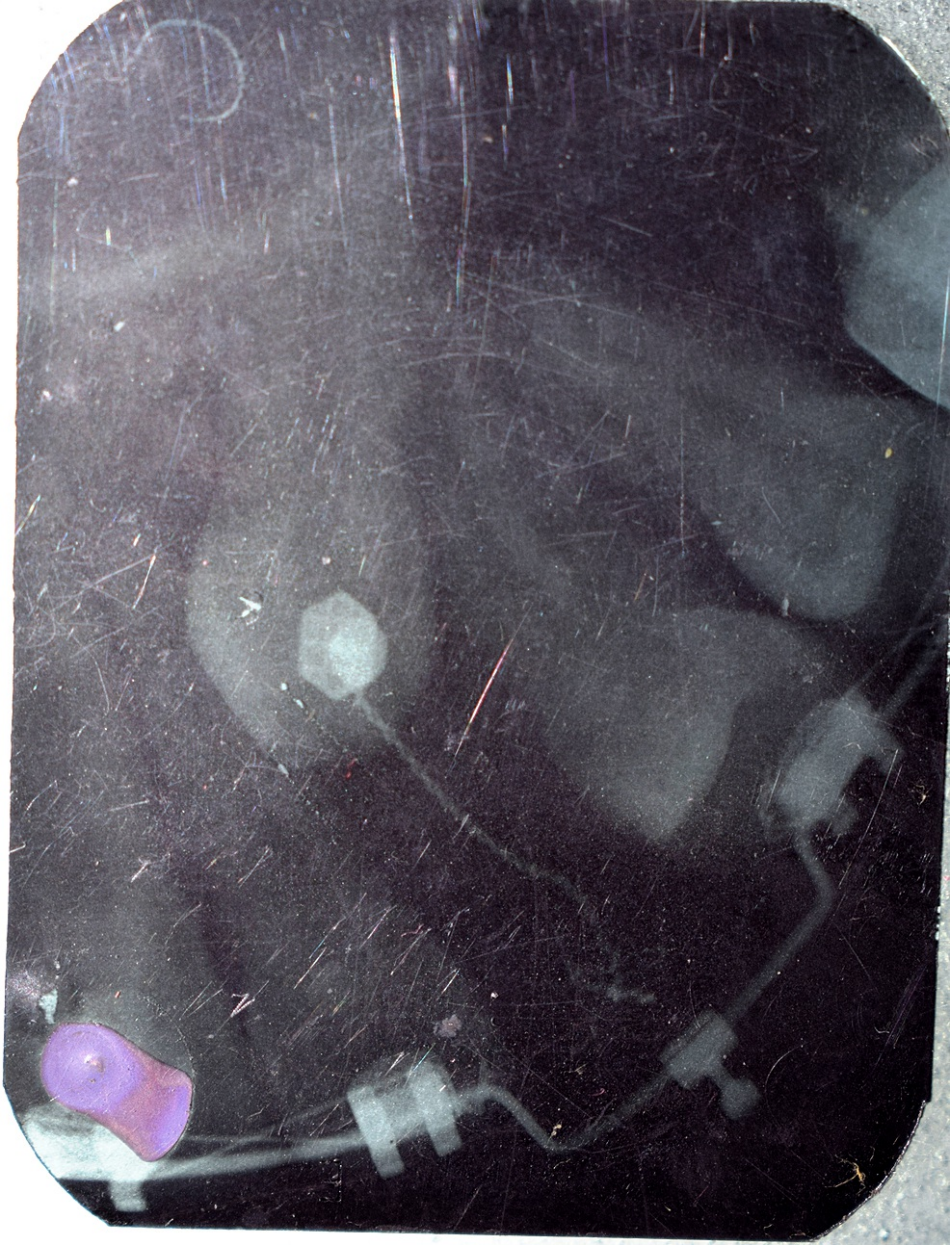
IOPA radiograph showing attachment position IOPA: intra-oral peri-apical

A 0.0011” ligature wire was placed on the lingual button and its ends were brought through the suture site. Canine traction was started using 120 g of elastic force from the end of the ligature wire to an attachment in the form of an inverted crimpable hook on the main 0.019 × 0.025” SS base archwire. The force applied was measured with a Dontrix gauge. Ligation with the figure of 8 wire was done from 16 to 22 and 24 to 27 to act as anchorage units. Forces applied for traction through elastics were changed at every appointment in the subsequent visits for the next eight months till canine emergence was seen (Figure 11).

**Figure 12:**
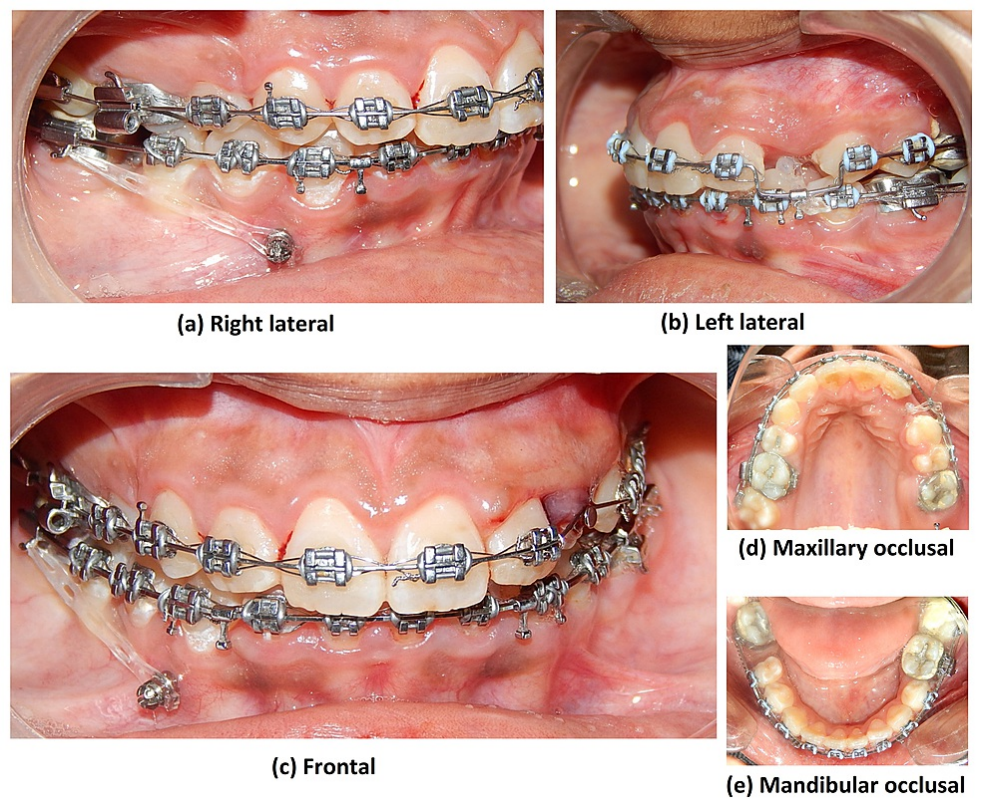
Intra-oral photograph views showing molar protraction with 47

Meanwhile, in the lower arch, TAD was placed between 43 and 44, and TAD-supported molar protraction (47) was started to close the existing space of missing 46 (Figure 12).

**Figure 13:**
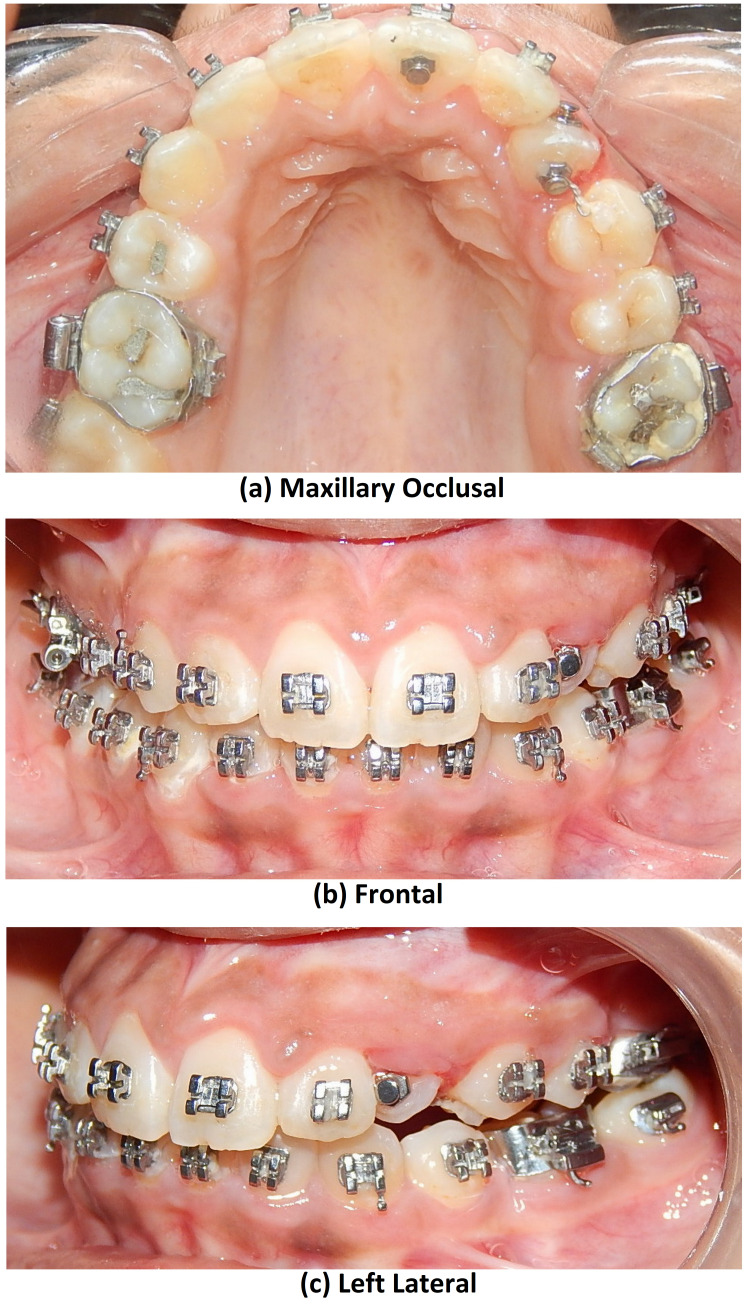
Intraoral photograph views showing canine emergence and derotation

After eight months of giving traction to bring the canine into the arch, canine emergence was seen in the oral cavity and the lingual button was attached to the buccal surface (Figure 13).

**Figure 14:**
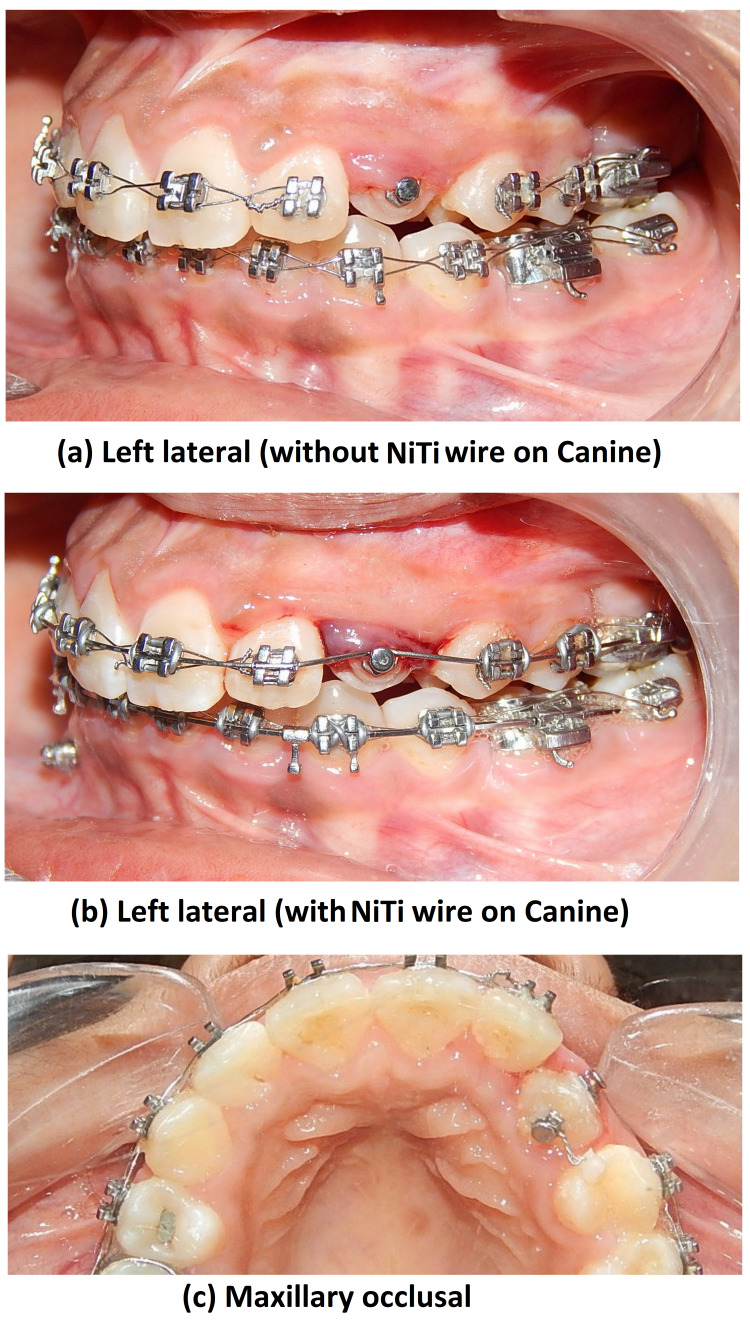
Intraoral photograph views showing derotated canine NiTi: nickel titanium.

As it was rotated, a couple of forces were applied for derotation. Post-derotation 0.14” NiTi was attached to make the canine erupt (Figure 14).

**Figure 15:**
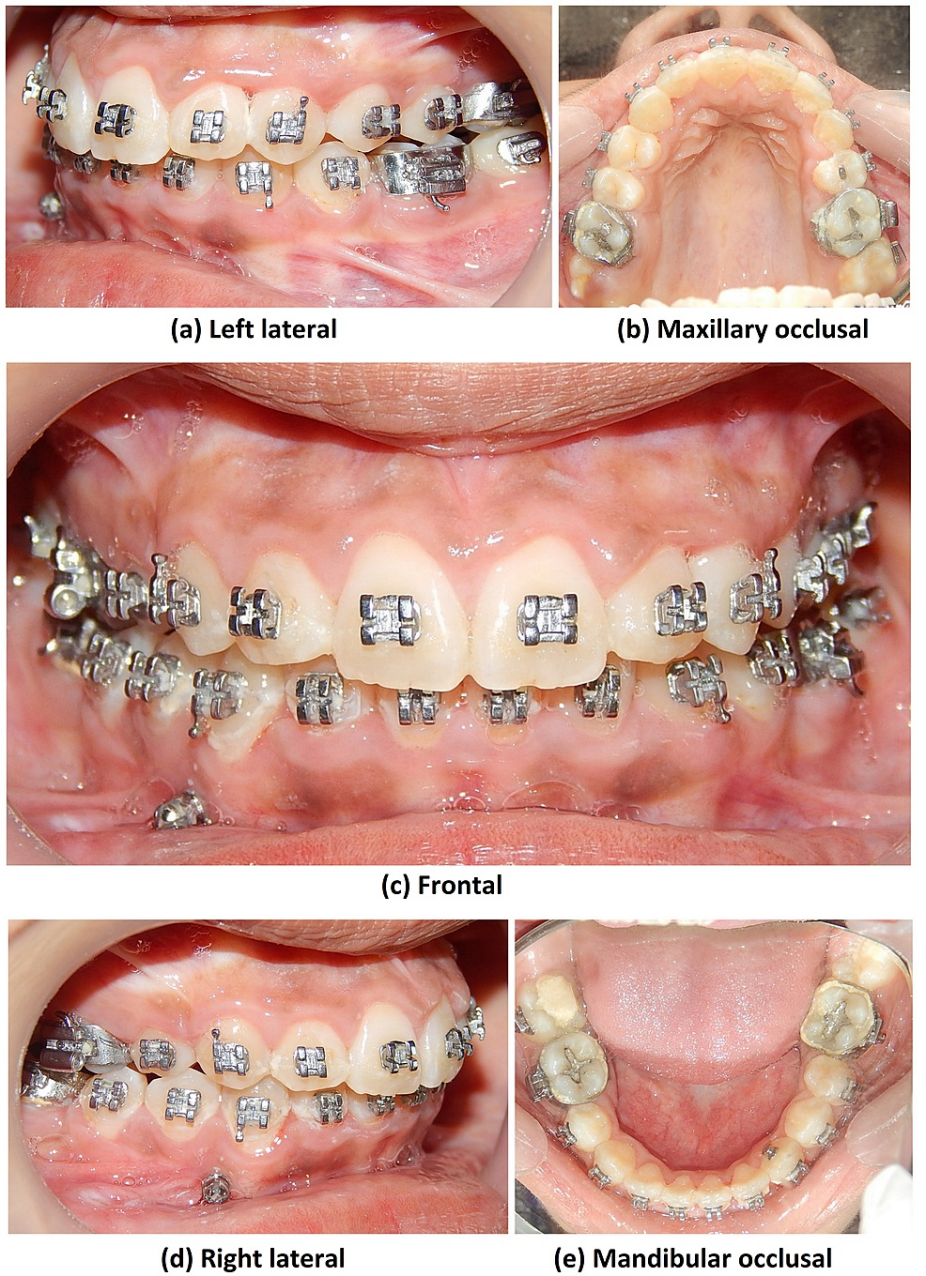
Pre-finishing intraoral photograph views

After two months, 23 was bonded and alignment of 23 was started using 0.014” NiTi wire. Post alignment, at three months on the 0.019 × 0.025” SS stage, a toe-out bend was given in the upper arch-wire along with cross elastics to correct posterior crossbite with 27. Crossbite correction took two months. Pre-finishing records were taken (Figure 15).

Settling was started and the case was debonded. The total treatment duration of this case was 24 months.

Treatment results

**Figure 16:**
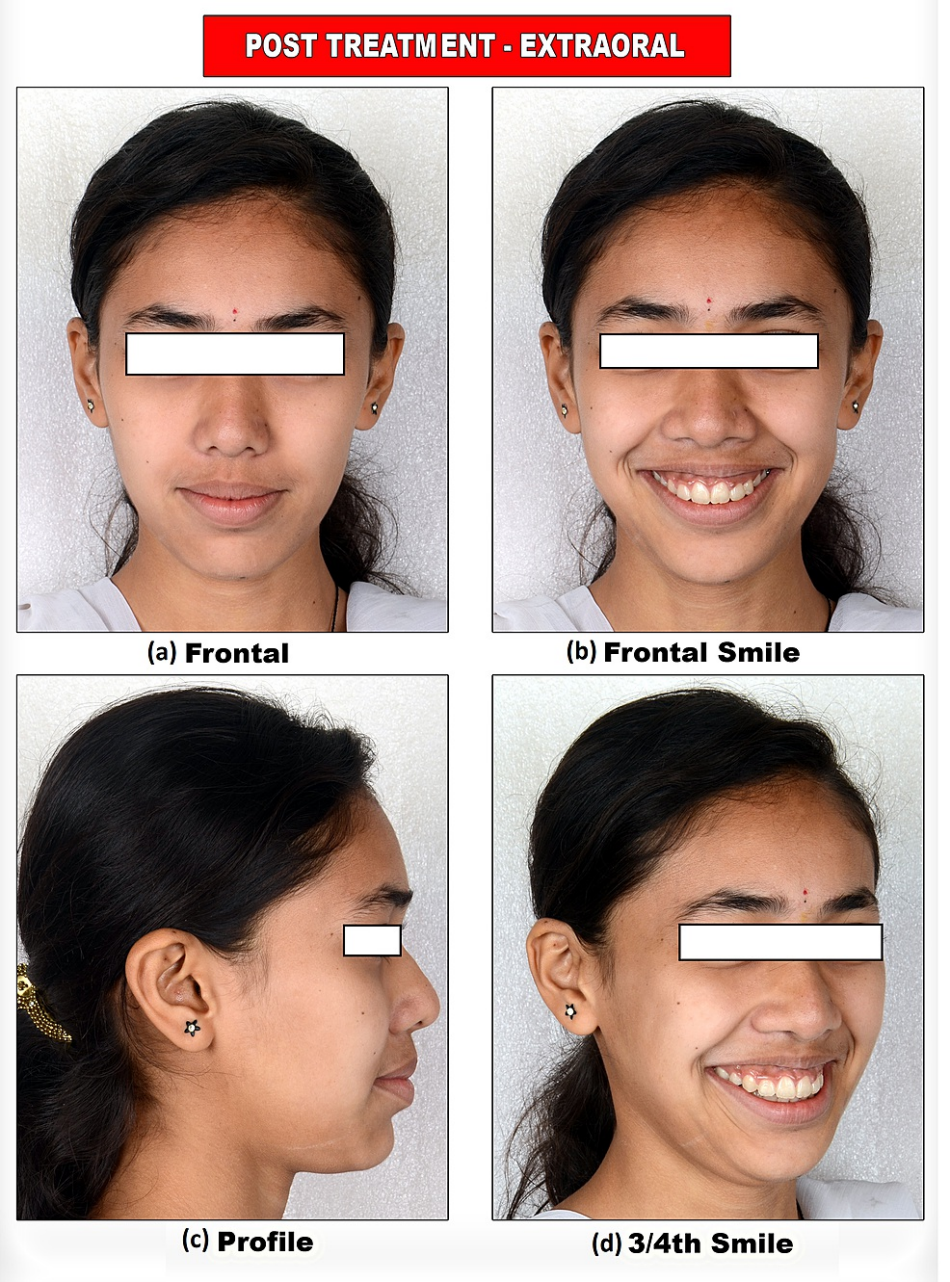
Post-treatment extraoral photograph views

After completion of the case, that patient’s chief complaint of irregularly placed and proclined anterior teeth was addressed. A consonant smile is obtained (Figure 16).

**Figure 17:**
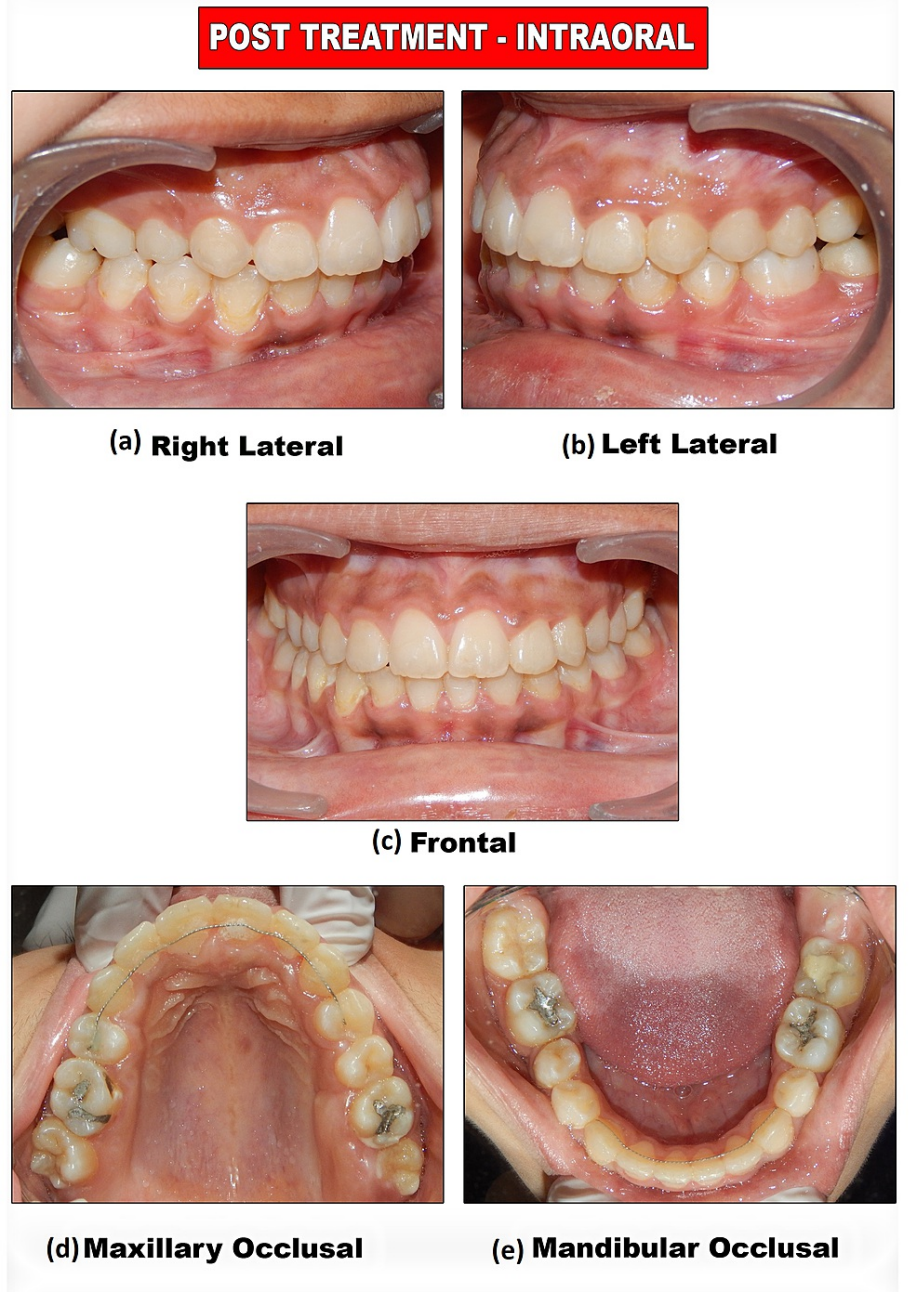
Post-treatment intraoral photograph views

Lip competency is achieved. Retraction of the upper anterior is achieved. The decrowding of upper and lower arches and the crossbite were corrected. The impacted canine was brought into the arch. Closure of lower missing molar spaces with molar protraction was achieved. Class I canine relations were obtained bilaterally. Satisfactory superclass II molar relations on the right and class III molar relations on the left occlusion with ideal overjet and overbite were also achieved with optimal facial aesthetics and balance (Figure 17).

## Discussion

Impaction of the maxillary canine is widely mentioned in the literature, and the reported incidence is 0.8-2.8% [3-5]. The exact etiology of canine impaction is unknown. Various authors have suggested different possible etiological factors. It has been suggested that the devious path the canine follows during its eruption and the long period of its development play an important role in its impaction [6]. A genetic or familial trend has been implicated as a cause of maxillary canine impaction, but in the present case, no familial background has been detected [7,8]. There have been many case reports in the literature for successful treatment of impacted canines but it is uncommon to have missing molars in the same case [9-13].

Major esthetic and/or functional disorders happen due to an impacted or unerupted tooth. Thus, there is most often a need to align it in the arch, especially when the impacted tooth is in the anterior region. For palatal impactions that are too severe, usually, surgical intervention is necessary that involves reflection of the palatal flap, followed by bone removal overlying the tooth to place an attachment on the tooth, followed by orthodontic traction [14,15]. With this in mind, treatment of tooth retention requires collaboration between surgeons and orthodontists to achieve maximum results and a long-term prognosis. Consequently, the introduction of surgical‑orthodontic techniques into our clinical practice has made it.

Adequate space should be created in the dental arch to allow for proper alignment of impacted teeth and later expose the tooth surgically so that a mechanical traction force can be applied to help erupt the tooth. It has been shown that many methods can efficiently create space for impacted teeth, but the easiest would be to use an open coil, which was used in this case.

The permanent first molar is the most important unit of mastication and for a functionally desirable occlusion. Loss of a first permanent molar can lead to changes in the dental arches. Unless appropriate corrective measures are instituted, these changes include diminished local function, tooth drifting, and continued eruption of opposing teeth. Also, molar protraction carried out with 47, in this case, helped to avoid a prosthesis as well as create space for the eruption of third molars.

During the treatment, implant-supported 27 retraction was carried out as anchor molar 27 could not be subjected to any force due to the critical space requirement in that quadrant. Surgical exposure and traction of the impacted 23 were done with a multidisciplinary approach, stabilizing the remaining arch. Periodic monitoring helped in evaluating the location of the impacted tooth.

**Figure 18:**
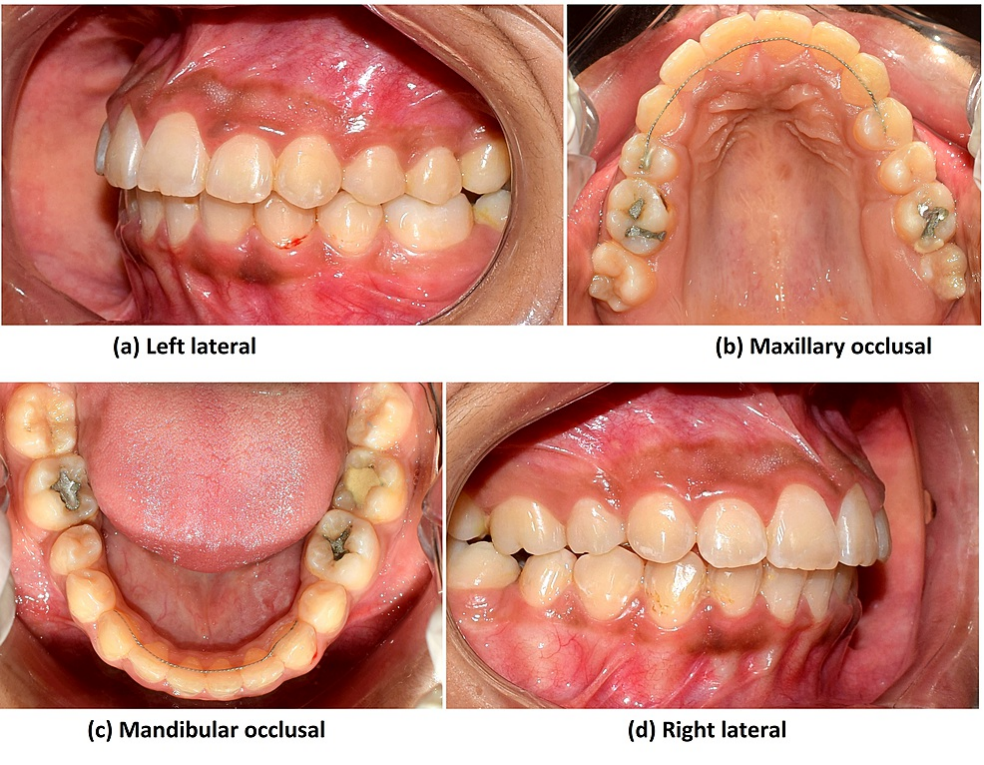
Two years post-retention intraoral photograph views

Post-treatment, ideal class 1 occlusion with molars was not achieved due to the missing molars; however, satisfactory superclass II molar relation on the right and class III molar relation on the left occlusion with ideal overjet and overbite was achieved. Also, class I canine relation was achieved, which is important for functional occlusion. A functionally stable cusp-embrasure relationship was achieved bilaterally. The achieved results were stable even at two years post-treatment (Figure 18).

## Conclusions

Thus, treatment options for any similar case need to be evaluated depending on factors like age, crowding, spacing, position and pathology of the impacted canine, considerations with missing contralateral molars (mutilated dentition), and other such clinical presentations. Though some patients hesitate for a detailed investigatory process, still, orthodontists should try to use the recent diagnostic techniques like CT scan which will provide a three-dimensional location of an impacted tooth. This will help with a better diagnosis and treatment plan. Also, an ideal molar relation is not always possible to achieve in cases of compromised teeth; hence, an attempt should be made to achieve a balanced and functional occlusion to the clinicians' best possible limits.
